# Ethnicity-Stratified Analysis of the Association between TNF-*α* Genetic Polymorphisms and Acute Kidney Injury: A Systematic Review and Meta-Analysis

**DOI:** 10.1155/2020/5262351

**Published:** 2020-10-05

**Authors:** Guanzhong Chen, Bowen Liu, Huanqiang Li, Ziling Mai, Liyao Zhang, Min Li, Liwei Liu, Shiqun Chen, Jiyan Chen, Yong Liu

**Affiliations:** ^1^Guangdong Provincial People's Hospital, School of Medicine, South China University of Technology, Guangzhou, 510000 Guangdong, China; ^2^Department of Cardiology, Guangdong Provincial Key Laboratory of Coronary Heart Disease Prevention, Guangdong Cardiovascular Institute, Guangdong Provincial People's Hospital, Guangdong Academy of Medical Sciences, Guangzhou, 510000 Guangdong, China; ^3^The Second School of Clinical Medicine, Southern Medical University, Guangzhou, 510515 Guangdong, China

## Abstract

**Background:**

Several studies have reported conflicting findings regarding the association between tumor necrosis factor-alpha (TNF-*α*) genetic polymorphisms and acute kidney injury (AKI). Therefore, we performed this meta-analysis to further investigate whether TNF-*α* variants are related to AKI susceptibility.

**Methods:**

A comprehensive search of observational studies on the association of TNF-*α* polymorphism with AKI susceptibility was conducted in the PubMed, Cochrane, and Embase databases through February 10, 2020. Pooled odds ratios (ORs) and 95% corresponding confidence intervals (95% CIs) were analyzed to evaluate the strength of the relationship.

**Results:**

A total of 8 studies involving 6694 patients (2559 cases and 4135 controls) were included. Pooled analysis showed a trend of increased risk between the TNF-*α* rs1800629 variant and AKI (A vs. G: OR [95%CI] = 1.33 [0.98‐1.81]) among the overall population. Ethnicity-stratified analysis indicated that the TNF-*α* rs1800629 variant was a risk factor for Asians (OR [95%CI] = 1.93 [1.59‐2.35]) while it is not for Caucasians (OR [95%CI] = 1.04 [0.91‐1.20]). Additionally, we also found that TNF-*α* rs1799964 polymorphism was observed to have a significant relationship with AKI risk in Asian patients (C vs. T, OR [95%CI] = 1.26 [1.11‐1.43]).

**Conclusions:**

The TNF rs1800629 polymorphism exhibited a trend toward AKI susceptibility with ethnic differences. The relationship was found to be significant among the Asian population, but not among those of Caucasian origin. Additionally, the TNF-*α* rs1799964 polymorphism was also related to a significantly increased risk of AKI in Asians.

## 1. Background

Acute kidney injury (AKI), characterized by a rapid decline in kidney function within two days, has become one of the most common syndromes in hospital settings worldwide [1]. AKI occurs in approximately 10–30% of patients admitted to the hospital. The rate is even as high as 50% when it comes to the high-risk patients [2, 3]. Furthermore, it is also associated with high mortality (10-30%) and a high occurrence of chronic kidney disease (24.6%) [2, 4]. The key step for preventing AKI is to identify high-risk patients [5]. Besides clinical risk factors [6, 7], genetic factors have been reported relative to interindividual differences in susceptibility to AKI [8, 9].

Tumor necrosis factor-*α* (TNF-*α*) could initiate the inflammatory cascade and induces the production of inflammatory mediators [10, 11]. Several TNF-*α* single nucleotide polymorphisms (SNPs), including rs1800629 and rs1799964, had been reported to associate with altered TNF-*α* expression [12, 13]. Some studies had analyzed the association between two TNF-*α* promoter SNPs (rs1800629 and rs1799964) and AKI susceptibility [14–18]. Dalboni et al. [14] and McBride et al. [14, 15] conducted studies in Caucasians and found that TNF-*α* rs1800629 had no association with susceptibility to AKI, while studies conducted in Asians indicated that both TNF-*α* rs1800629 and rs1799964 had a positive association with AKI [16, 18].

Even though several studies made an attempt to explore the genetic association between TNF-*α* and AKI risk [14–21], these studies had inconsistent results or small sample size. Therefore, we aimed to conduct this study to assess the genetic association as well as the ethnic difference of two TNF-*α* SNPs (rs1800629 and rs1799964) with AKI risk.

## 2. Methods

This work adheres to the Preferred Reporting Items for Systematic Reviews and Meta-Analyses (PRISMA) guidelines, and the protocol of this meta-analysis has been published on PROSPERO (https://www.crd.york.ac.uk/prospero/), of which the registration number is CRD42020191747.

### 2.1. Literature Search

A comprehensive strategy was used to search for studies about the correlation between TNF-*α* polymorphisms and AKI from the PubMed, Embase, and Cochrane databases. Searching terms or keywords were “Polymorphism, Genetic,” “Genetic,” “TNF-*α*,” “Tumor necrosis factor,” “AKI,” and “acute kidney injury.” The searched articles and abstracts were limited to those written in English before February 10, 2020 (for details, see Additional file 1).

### 2.2. Inclusion and Exclusion Criteria

Studies were included according to the following criteria: (1) evaluation of the association between TNF polymorphisms and AKI, (2) studies focusing on humans, and (3) studies with detailed genotype data. Studies were excluded if they were classified as (1) duplication of publications; (2) commentaries, reviews, editorials, animal studies, or case reports; (3) studies not relevant to TNF-*α* polymorphism and AKI risk; (4) studies about kidney transplant; or (5) studies lacking control groups or without detailed genotype data.

### 2.3. Quality Assessment

We used the Newcastle-Ottawa Scale (NOS) to evaluate the quality of the included studies. According to the scoring results, the included articles were classified as low, medium, and high quality, with scores of “1-3,” “4-5,” and “6-9,” respectively.

### 2.4. Data Extraction from the Selected Article

According to the inclusion and exclusion criteria, two reviewers selected articles by screening the title, abstract, and full text independently. Then, these two reviewers analyzed each paper and extracted detailed information. The following information was extracted: name of first author, publication year, SNPs, country, ethnicity, total sample size, numbers of cases and controls, and numbers of genotype frequencies. Any divergence was settled by a consensus of all authors.

### 2.5. Statistical Analysis

The Hardy–Weinberg equilibrium (HWE) was calculated for control groups of each study by using the *χ*^2^ test. The study population was considered a significant departure from genetic balance if *P* value < 0.05. Pooled ORs with 95% CIs were calculated for the allelic model (rs1800629: A vs. G, rs1799964: T vs. C), heterozygous model (rs1800629: GA vs. GG, rs1799964: TC vs. TT), homozygous model (rs1800629: AA vs. GG, rs1799964: CC vs. TT), dominant model (rs1800629: AA+GA vs. GG, rs1799964: CC+TC vs. TT), and recessive model (rs1800629: AA vs. GG+GA, rs1799964: CC vs. TT+TC). Heterogeneity was evaluated by the *Q* statistic and *I*^2^ statistic [22]. When *I*^2^ was >50%, a random-effect model was adopted. Otherwise, the fixed-effect model was used [23]. We also conducted a meta-regression analysis to determine the source of heterogeneity using a restricted maximum likelihood model with the Knapp-Hartung method [24, 25]. To assess the effect of ethnicity difference, we performed a subgroup analysis by ethnicity, categorized as Caucasian and Asian. Influence analysis was performed to recompute the pooled risk estimates for the remaining studies by removing each study successively.

Both Begg's test and Egger's test were used to assess publication bias [26]. Publication bias was considered with a significant difference when *P* < 0.05. STATA 12.0 software (Stata Corporation, TX, USA) was performed to do all statistical analyses.

## 3. Results

### 3.1. Characteristics of the Studies

**Figure 1:**
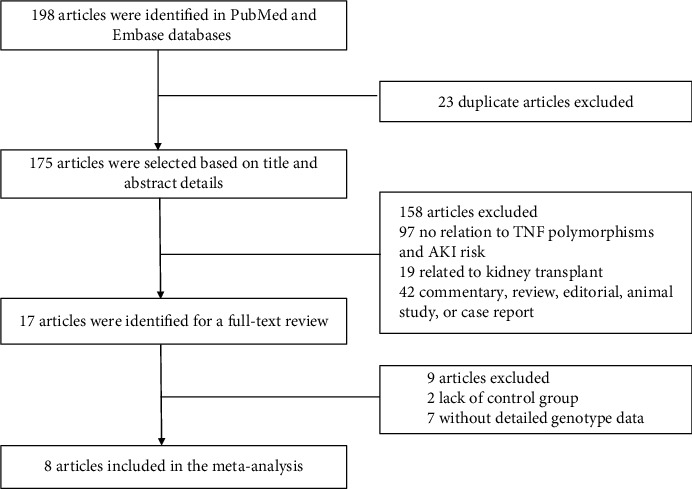
Flow chart of study selection.

The literature search strategy identified 198 related studies from the PubMed, Embase, and Cochrane databases. The literature selection process is shown in the schematic representation in Figure 1. A total of 181 articles were excluded by identifying the titles and abstracts, of which 23 were duplicates, 97 had no relation to this topic, 19 were related to kidney transplant, and 42 were commentaries, reviews, editorials, animal studies, or case reports. The remaining 17 studies were then reviewed in full text, and 9 studies were excluded, among which 2 lacked control groups and the others lacked detailed genotype data. Finally, 8 eligible studies [14, 15–21] including 6694 patients (2559 cases and 4135 controls) were chosen to be analyzed (Table 1). Within all of the included studies, there were 7 studies related to TNF-*α* rs1800629 polymorphism and 3 studies related to TNF-*α* rs1799964 polymorphism. Regarding the TNF-*α* rs1800629 studies, the subjects in 5 studies [14, 15, 19–21] were Caucasians and the subjects in the other 2 were Asians [16, 17]. Regarding the TNF-*α* rs1799964 studies, all the 3 included studies were conducted in the Asian population [16–18]. All the included studies were assessed as high quality (NOS score ≥ 6, see Additional file 2), and the genotyping distributions were genetically balanced (*P* > 0.05).

### 3.2. Meta-Analysis of TNF-*α* rs1800629

**Figure 2:**
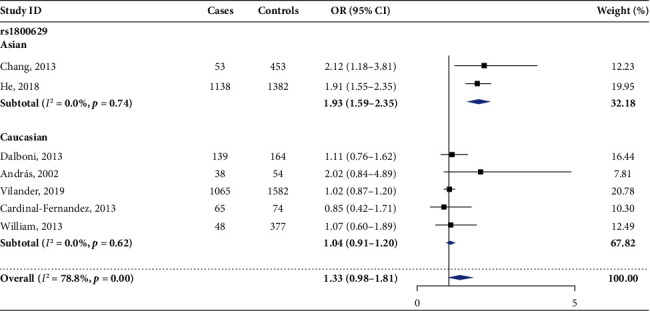
Forest plot of the allelic model of TNF-*α* rs1800629 (A vs. G).

In the pooled analysis of 7 eligible studies, significant heterogeneity was found in the allelic model (A vs. G), heterozygous model (AG vs. GG), and dominant model (AG+AA vs. GG); therefore, the random-effect model was used for these genetic models. Although there was no statistical significance found in the allelic model (OR [95%CI] = 1.33 [0.98‐1.81]), an increasing trend of AKI risk could be seen, as shown in Figure 2, while a significant association can be seen in the heterozygous comparison (OR [95%CI] = 1.43 [1.05‐1.96]) and dominant comparison (OR [95%CI] = 1.42 [1.02‐1.96]). Moreover, homozygous comparison (OR [95%CI] = 1.08 [0.72‐1.62]) and recessive comparison (OR [95%CI] = 1.03 [0.69‐1.53]) had no significance (see Table 2).

### 3.3. Meta-regression of TNF-*α* rs1800629

**Figure 3:**
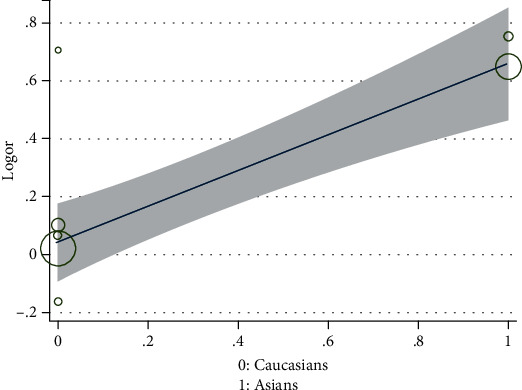
Meta-regression analysis of ethnicity for the allelic model of TNF-*α* rs1800629.

In order to explore the possible sources of heterogeneity, meta-regression analysis was performed. The result indicated that ethnicity had a significant association with outcomes at posttest (exp (*b*) = 1.85; *P* = 0.004; adjusted *R*^2^ = 100%, see Figure 3). To sum up, nearly most of heterogeneity could be explained by the ethnicity variance between included studies. However, sample size, case ratio, and sexual ratio were not significantly related to outcomes according to the results of the meta-regression (data not shown).

### 3.4. Ethnicity-Stratified Analysis of TNF-*α* rs1800629

We conducted a subgroup analysis by ethnicity. The ethnicity-stratified analysis had no significant heterogeneity for both Asian and Caucasian population analyses; thus, a fixed-effect model was employed. In Asians, all the five genetic models could be detected to significantly associate with AKI susceptibility (see Table 2). However, in Caucasians, there was no significant correlation in any genetic model (see Table 2).

### 3.5. Meta-Analysis of TNF-*α* rs1799964

**Figure 4:**
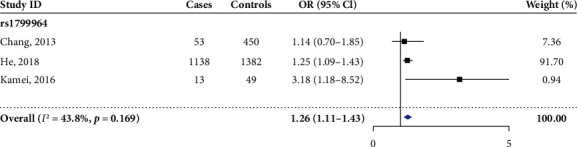
Forest plot of the allelic model of TNF-*α* rs1799964 (C vs. T).

A total of 3 studies were taken into the meta-analysis of the correlation between the TNF-*α* rs1799964 and AKI susceptibility. All the included studies were carried out in Asian populations; therefore, no significant heterogeneity was identified. The pooled analysis showed that the rs1799964 T allele was associated with the increased risk of AKI in all the genetic models (see Figure 4, Table 2, allelic comparison: OR [95%CI] = 1.26 [1.11‐1.43]; heterozygous comparison: OR [95%CI] = 1.18 [1.00‐1.39]; homozygous comparison: OR [95%CI] = 1.82 [1.28‐2.59]; dominant comparison: OR [95%CI] = 1.25 [1.07‐1.46]; and recessive comparison: OR [95%CI] = 1.72 [1.22‐2.44]).

### 3.6. Influence Analysis

**Figure 5:**
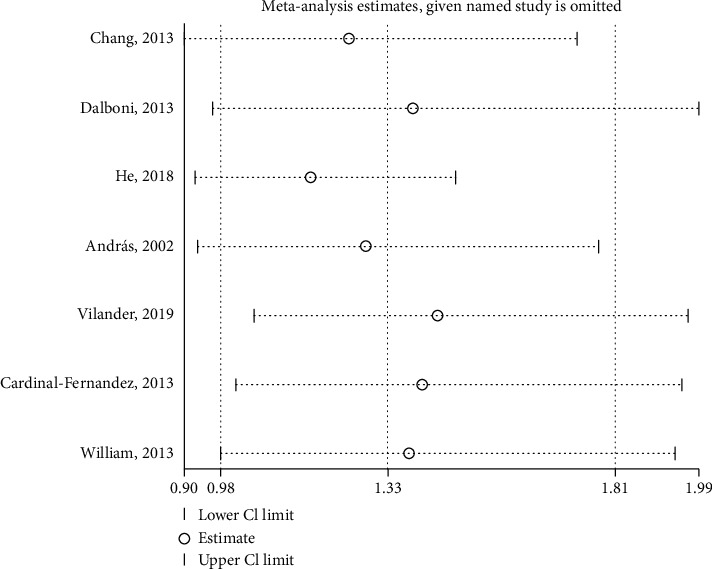
Influence analysis of the TNF-*α* rs1800629 allelic model (A vs. G).

Influence analysis recomputing for the remaining studies by removing each study successively contributed to little change on the pooled results, from OR [95%CI] = 1.17 [0.93‐1.48] to OR [95%CI] = 1.44 [1.05‐1.97] (see Figure 5).

### 3.7. Publication Bias

**Figure 6:**
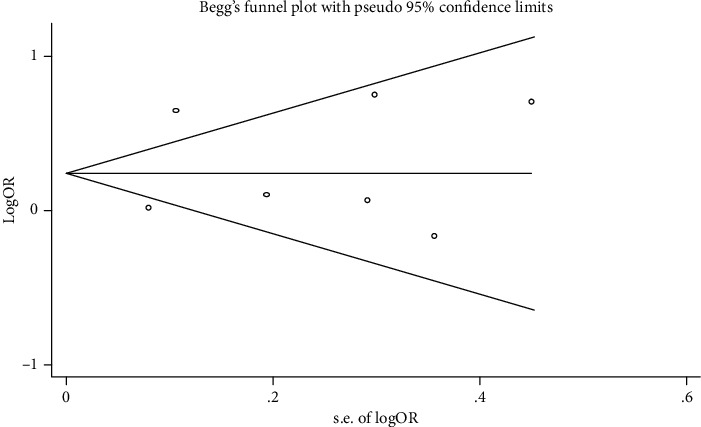
Funnel plot for publication bias analysis of TNF-*α* rs1800629 (A vs. G).

Publication bias was evaluated by Begg's funnel plot and Egger's regression test. The results showed that no significant publication bias was found in the TNF-*α* rs1800629 variant (Begg's test: *P* = 0.764; Egger's test: *P* = 0.722). Symmetrical funnel plots are shown in Figure 6. Owing to the limited included studies, no publication bias test was conducted for the TNF-*α* rs1799964.

### 3.8. SNP Allele Frequency in Populations

As shown above, TNF-*α* rs1800629 was significantly associated with AKI susceptibility in Asians and but not in Caucasians. We next investigated the allele frequencies of TNF-*α* rs1800629 between ethnicity (see Table 3). In our meta-analysis, the rs1800629 A allele frequency in the control group (0.149 for Caucasians, 0.063 for Asians) was similar with that in the 1000 Genomes Project (0.134 for Europeans, 0.059 for Asians). However, TNF-*α* rs1799964 C allele frequency in the control group (0.133 for Asians) was different from that in the 1000 Genomes Project (0.196 for Asians).

## 4. Discussion

To our knowledge, the present study is the first global systematic meta-analysis to evaluate the genetic relationship between TNF-*α* polymorphisms and AKI susceptibility. The results indicated that TNF-*α* rs1800629 had a trend of increased risk of AKI for all patients. What is more, this association was significant in Asian patients, but was not significant in Caucasian patients by subgroup analysis. Additionally, because of the limited number of included studies, TNF-*α* rs1799964 was shown to be related to an increased risk for AKI only in Asian patients.

In the previous systematic reviews, both Vilander et al. [8] and Larach et al. [9] indicated that the relationship between TNF-*α* polymorphisms and AKI susceptibility was controversial. In comparison, both of them did not assess the effect of TNF-*α* polymorphisms or conduct a detailed analysis of the conflicting results. Consequently, we performed this meta-analysis to quantificationally evaluate the relationship between TNF-*α* polymorphisms and AKI susceptibility. More importantly, we found that TNF-*α* polymorphisms had a trend of increasing the risk of AKI susceptibility with ethnic differences. In the present meta-analysis, the association between TNF-*α* rs1800629 and AKI susceptibility was not significant for the overall populations. Meta-regression and ethnicity-stratified analyses were performed to investigate the main source of heterogeneity. The results showed that the association of TNF-*α* rs1800629 with AKI susceptibility varied by ethnicity. What is more, the subgroup results indicated that the rs1800629 GA/AA genotype had a higher risk of AKI among Asians but not among Caucasians. Several possible explanations may account for these findings. One of the reasons is that the studies conducted in Caucasians mainly included critically ill patients from the intensive care unit with severe infection [14, 19–21] or underwent cardiac surgery [15]. The patients included in Asian studies underwent coronary artery intervention [17] or were pediatric patients [16], with a lower incidence of comorbidity or better baseline renal function. Another possible explanation is that the distribution of TNF-*α* rs1800629 alleles is population dependent. In the present meta-analysis, the frequencies of the rs1800629 A allele in the control group were 6.26% for Asians [16, 17] and 14.90% for Caucasians [14, 15, 19–21]. This result was similar to that of the 1000 Genomes Project (as shown in Table 3) and that reported by Zhang et al. (8.20% for Asians and 15.70% for Caucasians) [27]. Moreover, several meta-analysis studies also indicated that the rs1800629 A allele had a significant association with susceptibility to osteoarthritis [28], dilated cardiomyopathy [27], and chronic obstructive pulmonary disease [29] among the Asian population, but not among the Caucasian population. These findings indicated that the dependent distribution of alleles could partially explain the susceptibility differences in the ethnic background.

Regarding TNF-*α* rs1799964, a total of 3 eligible studies conducted in the Asian population including 1204 cases and 1881 controls were analyzed. Although there was a difference of the TNF-*α* rs1799964 allele frequency observed between our meta-analysis and the 1000 Genomes Project data, there was no deviation from HWE in the control group of all included studies. The difference may be due to the small sample size or the heterogeneity between the included studies. The relationship between TNF-*α* rs1799964 and AKI susceptibility was found in all the genetic comparisons, suggesting that the rs1799964 variant might lead to a higher risk for AKI in the Asian population, while the relationship needs to be verified in a larger sample size.

Nevertheless, some limitations of this study should not be ignored. First, it is better to further evaluate the effect of TNF-*α* polymorphisms on AKI susceptibility by screening in the online database, while it is difficult for us to get access to the raw data of AKI genomics to make the validation. Second, AKI is a multifactorial disease. Some confounding factors such as gender, history of nephrotoxic drugs, and heart function may be addressed across the included studies. However, we have no access to get these clinical data to estimate the effect of confounding factors. Third, significant heterogeneity was found in the rs1800629 analysis for the overall population. Thus, we performed meta-regression and ethnicity subgroup analyses to investigate the source of the heterogeneity. Last, the included studies for TNF-*α* rs1800629 subgroup analysis or rs1799964 analysis were relatively limited; the results of these analyses should be interpreted prudently.

## 5. Conclusions

In conclusion, TNF rs1800629 polymorphism had a trend of increasing the risk of AKI with ethnic differences. The trend was significant among Asian patients, but was not among Caucasian patients. Additionally, the TNF-*α* rs1799964 polymorphism was related to a significantly increased risk of AKI in Asians. However, as AKI is a multifactorial disease, our findings require verification in larger samples and functional studies in the future.

## Figures and Tables

**Table 1:** Characteristics of studies included in the meta-analysis.

First author, year	Country	Ethnicity	Number (cases/controls)	Case			Control			*P* for HWE^∗^	Quality score
TNF-*α* rs1800629 G>A				GG	GA	AA	GG	GA	AA		
Chang, 2013	China	Asian	53/453	39	12	2	387	62	4	0.393	8
Dalboni, 2013	Brazil	Caucasian	139/164	76	60	3	102	52	10	0.339	7
He, 2018	China	Asian	1138/1382	309	219	10	1226	152	4	0.756	7
András, 2002	Hungary	Caucasian	38/54	25	13	0	44	10	0	0.453	8
Vilander, 2019	Finland	Caucasian	1065/1582	781	260	24	1171	373	38	0.209	7
Cardinal-Fernandez, 2013	Spain	Caucasian	65/74	50	14	1	54	19	1	0.640	7
William, 2013	England	Caucasian	48/377	33	14	1	271	93	13	0.162	7
TNF-*α* rs1799964 T>C				TT	TC	CC	TT	TC	CC		
Chang, 2013	China	Asian	53/450	33	16	4	285	146	19	0.956	8
He, 2018	China	Asian	1138/1382	676	395	67	894	437	51	0.790	7
Kamei, 2016	Japan	Asian	13/49	7	3	3	37	10	2	0.243	8

^∗^HWE: Hardy–Weinberg equilibrium.

**Table 2:** Summary of pooled ORs in the meta-analysis.

Comparison model	Studies	Overall effect		Heterogeneity	
		OR (95% CI)	*P* value	I^2^	*P* value
*TNF-α rs1800629*					
Allelic model (A vs. G)					
Overall	7	1.33 (0.98-1.81)	0.066	78.8%	<0.001
Asian	2	1.93 (1.59-2.35)	<0.001	0.0%	0.737
Caucasian	5	1.04 (0.91-1.20)	0.529	0.0%	0.617
Heterozygous model (GA vs. GG)					
Overall	7	1.43 (1.05-1.96)	0.025	72.7%	0.001
Asian	2	1.94 (1.57-2.40)	<0.001	0.0%	0.975
Caucasian	5	1.18 (0.92-1.51)	0.188	24.0%	0.261
Homozygous model (AA vs. GG)					
Overall	7	1.08 (0.72-1.62)	0.705	46.3%	0.097
Asian	2	3.65 (1.34-9.92)	0.011	0.0%	0.709
Caucasian	5	0.82 (0.52-1.30)	0.408	0.0%	0.682
Dominant model (AA+GA vs. GG)					
Overall	7	1.42 (1.02-1.96)	0.036	76.0%	<0.001
Asian	2	1.99 (1.62-2.46)	<0.001	0.0%	0.864
Caucasian	5	1.10 (0.93-1.30)	0.289	3.2%	0.389
Recessive model (AA vs. GA+GG)					
Overall	7	1.03 (0.69-1.53)	0.898	46.0%	0.099
Asian	2	3.30 (1.22-8.93)	0.019	0.0%	0.724
Caucasian	5	0.79 (0.50-1.25)	0.318	0.0%	0.543
*TNF-α rs1799964*					
Allelic model (C vs. T)					
Overall (Asian)	3	1.26 (1.11-1.43)	<0.001	43.8%	0.169
Heterozygous model (TC vs. TT)					
Overall (Asian)	3	1.18 (1.00-1.39)	0.045	0.0%	0.727
Homozygous model (CC vs. TT)					
Overall (Asian)	3	1.82 (1.28-2.59)	0.001	9.8%	0.330
Dominant model (CC+TC vs. TT)					
Overall (Asian)	3	1.25 (1.07-1.46)	0.005	0.0%	0.431
Recessive model (CC vs. TC+TT)					
Overall (Asian)	3	1.72 (1.22-2.44)	0.002	8.1%	0.337

**Table 3:** Allele frequency comparison between meta-analysis and the 1000 Genomes Project.

Polymorphisms	Ethnicity	Meta-analysis (allele frequencies)				1000 Genomes Project (allele frequencies)	
		Case		Control			
rs1800629		G	A	G	A	G	A
	Caucasian	0.845	0.155	0.851	0.149	0.866 (EUR)	0.134 (EUR)
	Asian	0.784	0.216	0.937	0.063	0.942 (EA)	0.059 (EA)
rs1799964		T	C	T	C	T	C
	Asian	0.767	0.233	0.868	0.133	0.805 (EA)	0.195 (EA)

EUR: European; EA: East Asian.

## Data Availability

The data used and/or analyzed during the current study are available from the corresponding author on reasonable request.

## Supplementary Materials

Supplementary Materials Additional file 1: detailed strategy of the literature search. Additional file 2: quality assessment of the included studies using the Newcastle-Ottawa Scale.
