# Supplementary material for: Ethnicity-Stratified Analysis of the Association between TNF-α Genetic Polymorphisms and Acute Kidney Injury: A Systematic Review and Meta-Analysis
Source: Biomed Res Int. 2020 Oct 5;2020:5262351. doi: 10.1155/2020/5262351 (PMC7556080; doi:10.1155/2020/5262351)
Supplement: Supplementary Materials — Additional file 1: detailed strategy of the literature search. Additional file 2: quality assessment of the included studies using the Newcastle-Ottawa Scale. [file 5262351.f1.docx]

**Supplementary information**

Additional file 1. Detailed strategy of the literature search.

Additional file 2. Quality assessment of the included studies using the Newcastle-Ottawa Scale.

| **Databases** | **Detailed strategy** |
| --- | --- |
| **PubMed** | ((((((((((((((Tumor Necrosis Factor alpha[MeSH Terms]) OR Cachectin) OR Cachectin-Tumor Necrosis Factor) OR Cachectin Tumor Necrosis Factor) OR Tumor Necrosis Factor Ligand Superfamily Member 2) OR Tumor Necrosis Factor) OR TNF Superfamily, Member 2) OR TNFalpha) OR TNF-alpha) OR TNF-alpha) OR TNFalpha) OR tumor necrosis factor-alpha)) AND (((((("acute kidney injury"[MeSH Terms] OR acute kidney injury[All Fields]) OR (acute kidney injury[All Fields] OR acute kidney injury,[All Fields]) OR (acute[All Fields] AND kidney failure[All Fields]) OR ((Acute[All Fields] AND ("kidney"[MeSH Terms] OR kidney[All Fields])) AND (insufficiencies[All Fields] OR insufficiency[All Fields])) OR AKI[All Fields] OR ARF[All Fields] OR (acute renal injury[All Fields] OR acute renal injury,[All Fields]) OR acute renal failure[All Fields] OR acute renal insufficiency[All Fields]) OR (exp[All Fields] AND ("renal insufficiency"[MeSH Terms] OR renal insufficiency[All Fields]))) OR ((exp[All Fields] AND ("nephritis"[MeSH Terms] OR nephritis[All Fields])) OR (acute[All Fields] AND ("kidney diseases"[MeSH Terms] OR Kidney Diseases[All Fields]))) OR ((acute[All Fields] AND ("nephritis"[MeSH Terms] OR nephritis[All Fields])) OR (acute[All Fields] AND (nephropathic[All Fields] OR nephropathies[All Fields] OR nephropathologists[All Fields] OR nephropathy[All Fields])) OR (acute[All Fields] AND nephrotoxic$[All Fields])))))) AND (((((((((((genomic[Title/Abstract] OR genotype[Title/Abstract]) OR polymorphism[Title/Abstract]) OR single nucleotide polymorphism[Title/Abstract]) OR single nucleotide variant[Title/Abstract]) OR mutation[Title/Abstract]) OR polymorphisms[Title/Abstract]) OR GWAS[Title/Abstract] OR Genome-wide association[Title/Abstract] OR association study[Title/Abstract])) OR ((GWAS[Title/Abstract] OR Genome-wide association study[Title/Abstract]))))) |
| **Embase** | (aki OR 'acute kidney failure' OR 'kidney injury') AND ('tumor necrosis factor' OR tnf) AND ('genetic polymorphism' OR 'dna polymorphism' OR 'single nucleotide polymorphism' OR 'genetic variability' OR variant) |
| **Cochrane** | ("TNF alpha"):ti,ab,kw AND (acute kidney injury):ti,ab,kw AND (polymorphisms):ti,ab,kw |

**Additional file 1.** Detailed strategy of the literature search.

**Additional file 2.** Quality assessment of the included studies using the Newcastle-Ottawa Scale.

| **Study** | **Selection** | | | | **Comparability** | | **Exposure** | | | **Quality Score** |
| --- | --- | --- | --- | --- | --- | --- | --- | --- | --- | --- |
|  | **Adequate definition of case** | **Representativeness of the cases** | **Selection of controls** | **Definition of controls** | **Comparable**  **for 1,2,3*** | **Comparable**  **for 4,5,6,7^+^** | **Ascertainment of exposure** | **Same method of**  **ascertainment** | **non-response**  **rate** |  |
| Chang, 2013 | 1 | 1 | 1 | 1 | 1 | 1 | 1 | 1 | 0 | 8 |
| Dalboni, 2013 | 1 | 1 | 1 | 1 | 0 | 1 | 1 | 1 | 0 | 7 |
| He, 2018 | 1 | 1 | 1 | 1 | 1 | 1 | 1 | 1 | 0 | 8 |
| András, 2002 | 1 | 1 | 1 | 1 | 0 | 1 | 1 | 1 | 0 | 7 |
| Vilander，2019 | 1 | 1 | 1 | 1 | 0 | 1 | 1 | 1 | 0 | 7 |
| Cardinal, 2013 | 1 | 1 | 1 | 1 | 0 | 1 | 1 | 1 | 0 | 7 |
| William T, 2013 | 1 | 1 | 1 | 1 | 0 | 1 | 1 | 1 | 0 | 7 |
| Kamei, 2016 | 1 | 1 | 1 | 1 | 0 | 0 | 1 | 1 | 0 | 6 |

Comparability variables: 1 = age; 2 = sex; 3 = baseline kidney function; 4 = sepsis; 5 = diabetes mellitus history; 6 = heart failure history; 7 = hypertension history.

* If all the characteristics were comparable, a score was obtained; otherwise, no score was provided.

+ If one of the characteristics was comparable, a score was obtained; otherwise, no score was provided.
